# Environmental and occupational respiratory diseases – 1039. Clinical course and side effects of anti-IgE monoclonal antibody in patients with severe persistent asthma

**DOI:** 10.1186/1939-4551-6-S1-P38

**Published:** 2013-04-23

**Authors:** Arzu Didem Yalcin, Atil Bisgin, Ramazan Cetinkaya, Reginald M Gorczynski

**Affiliations:** 1 Internal Medicine, Allergy and Immunology, Education and Research Hospital, Turkey; 2 Cancer Institue, Sweden; 3 Department of Internal Medicine, Antalya Education and Research Hospital, Antalya,, Turkey; 4 Division of Cellular & Molecular Biology, Toronto Hospital, University Health Network, Toronto, ON, Canada

## Background

Omalizumab, a recombinant humanized monoclonal antibody to IgE, is recommended as a new option for the treatment of severe persistent allergic asthma. The purpose of this study is to assess the effects omalizumab treatment on life quality and its side effects in the severe persistent asthma patients.

## Methods

In this study, we evaluated 19 severe persistent asthma patients who received therapy with omalizumab for 8 months. Omalizumab was administered every 2 weeks between the doses of 150 to 375 mg. Symptoms and severity of allergic reactions were recorded before and after being on omalizumab. IgE levels, mean platelet volume (MPV), platelet levels, pulmonary function test and asthma control test were evaluated in all patients before and 8 months after the treatment. Local and systemic side effects of omalizumab were evaluated. Stool parasites were examined at 4th and 8th months after initiation of treatment to investigate any parasitosis.

## Results

The patients had severe persistent asthma for periods ranging from 3 to 8 years, and they were diagnosed with allergic asthma for 7-28 years. Thrombocytopenia developed in a male patient after the 22nd dose of the drug was given. When the platelet count fell down to 55.000, the omalizumab treatment was suspended. During the therapy period, one patient had parasitosis (giardiasis), one patient had severe side effects, one patient had dyspnea two hours after the injection, and one patient had a dyspnea attack 2 hours after the injection. The changes in MPV levels were not statistically significant. There was also significant decrease in IgE levels after the treatment.

## Conclusions

Our clinical follow-up study suggesting that monitoring the complete blood cell count might be important in the use of omalizumab. Only in one case, a dyspnea attack occurred 2 hours after the injection. The patient was hospitalized and treated appropriately, and discharged after a 24 hours of follow-up period. Thrombocytopenia developed in one patient and the treatment was suspended. Although we did not have any anaphylaxis, we believe that the patients should be monitored at least for 3 hours after the Omalizumab injection.

